# Relationship between Regional Fat Distribution and Hypertrophic Cardiomyopathy Phenotype

**DOI:** 10.1371/journal.pone.0158892

**Published:** 2016-07-07

**Authors:** Valeria Guglielmi, Luciano Maresca, Chiara Lanzillo, Giorgia Michela Marinoni, Monica D’Adamo, Mauro Di Roma, Paolo Preziosi, Alfonso Bellia, Leonardo Calò, Paolo Sbraccia

**Affiliations:** 1 Department of Systems Medicine, University of Rome “Tor Vergata”, Rome, Italy; 2 Diagnostic Imaging Department, Policlinico Casilino, Rome, Italy; 3 Cardiology Department, Policlinico Casilino, Rome, Italy; University of Bologna, ITALY

## Abstract

**Background:**

Hypertrophic cardiomyopathy (HCM), the most common genetic heart disease, is characterized by heterogeneous phenotypic expression. Body mass index has been associated with LV mass and heart failure symptoms in HCM. The aim of our study was to investigate whether regional (trunk, appendicular, epicardial) fat distribution and extent could be related to hypertrophy severity and pattern in HCM.

**Methods:**

Cardiovascular magnetic resonance was performed in 32 subjects with echocardiography-based diagnosis of HCM (22M/10F, 57.2±12.6 years) characterized by predominant hypertrophy at the interventricular septum (IVS). Regional fat distribution was assessed by dual-energy X-ray absorptiometry.

**Results:**

Gender differences were detected in maximum IVS thickness (M: 18.3±3.8 mm vs. F: 14.3±4 mm, p = 0.012), right ventricle (RV) systolic function (M: 61.3±6.7%; F: 67.5±6.3%, p = 0.048), indexed RV end-diastolic (M: 64.8±16.3 ml/m^2^; F: 50.7±15.5 ml/m^2^, p = 0.04) and end-systolic volumes (M: 24.3±8.3 ml/m^2^; F: 16.7±7.4 ml/m^2^, p = 0.04). After adjusting for age and gender, maximum IVS thickness was associated with truncal fat (Tr-FAT) (β = 0.43, p = 0.02), but not with either appendicular or epicardial fat. Epicardial fat resulted independently associated with NT-proBNP levels (β = 0.63, p = 0.04). Late Gadolinium Enhancement-positive subjects displayed greater maximum IVS thickness (p = 0.02), LV mass index (p = 0.015) and NT-proBNP levels (p = 0.04), but no associations with fat amount or distribution were observed.

**Conclusion:**

Truncal, but not appendicular or epicardial fat amount, seems to be related with maximum IVS thickness, the hallmark feature in our cohort of HCM patients. Further prospective researches are needed to assess a potential causative effect of central adiposity on HCM phenotype.

## Introduction

Hypertrophic cardiomyopathy (HCM), the most heterogeneous cardiac disease in terms of phenotypic expression and clinical outcome, represents the most common inherited cardiomyopathic process with an autosomal dominant trait of inheritance [1, 2]. In the vast majority of genotype-positive patients, HCM is associated with mutations in genes encoding proteins of the cardiac sarcomere, most commonly beta-myosin heavy chain and myosin-binding protein C [3–5]. The distribution of left ventricular (LV) hypertrophy, the anatomic hallmark of HCM [6], considerably varies in extent and distribution. Hypertrophy is typically asymmetric and involves the interventricular septum (IVS), but can involve any other segment of the LV [6, 7] and may occasionally be extended to the right ventricle (RV). Different genetic causes of HCM do not correlate with the pattern of hypertrophy, with a few exceptions such as troponin T mutations that generally cause milder hypertrophy [8], or an unique actin gene mutation which produces apical hypertrophy [9].

Increased body mass index (BMI) was recently reported to influence disease expression and clinical course in patients with HCM [10]. In addition, there is growing evidence that epicardial adipose tissue (EAT), which is characteristically more expanded in obese individuals with overweight-related metabolic derangements [11], may substantially affect both myocardial morphology and function, irrespective of the presence or not of a primitive cardiomyopathy [12]. In accordance, EAT was found to be related with LV mass and impaired diastolic function [13], as well as with myocardial fibrosis [14] and triglycerides content [15].

To our knowledge, the previous studies examining potential relationships between adiposity measures [16, 17] and LV morphology have not addressed the relation of body fat distribution with phenotypic expression and heart failure symptoms in primary genetic cardiomyopaties.

Thus, aim of our study was to investigate whether regional (trunk, appendicular and epicardial) fat distribution and extent are associated with pattern and severity of cardiac hypertrophy in adult overweight individuals with HCM.

## Materials and Methods

### Study population

We enrolled 32 consecutive adult patients (22 males, 10 females, age 57.2±12.6 years) who referred to the Cardiology Unit of Policlinico Casilino of Rome (Italy) between 2013 and 2014 and were diagnosed with HCM.

Diagnosis of HCM was based on 2-dimensional echocardiographic evidence of a non-dilated and hypertrophied left ventricle (wall thickness ≥15 mm in one or more LV myocardial segments), in the absence of another cardiac or systemic disease that could explain the magnitude of hypertrophy. For the purpose of the present study, we selectively included individuals with preferential localization of hypertrophy within the interventricular septum (IVS), in order to study a phenotypically homogeneous cohort of HCM patients. In case of lesser degrees of wall thickening (13–14 mm), the diagnosis of HCM required further evaluation of other features including family history, non-cardiac symptoms and signs, electrocardiogram (ECG) abnormalities, laboratory tests and multi-modality cardiac imaging [18].

In hypertensive patients, diagnosis of HCM was based on at least one of the following criteria: known HCM-causing sarcomere gene mutations or family history of HCM; onset of hypertension years after the diagnosis of HCM; maximum LV wall thickness exceeding that expected by hypertension alone (>20 mm); presence of marked mitral leaflet elongation [19]; LV outflow obstruction (≥30 mmHg) [20]; and distribution of late gadolinium enhancement (LGE) in CMR consistent with HCM (primarily mid-wall or transmural, and not confined to a single coronary vascular territory) [21, 22]. In first degree adult relatives of patients with HCM the diagnosis was fulfilled by the presence of one major or two minor echocardiographic criteria, or one minor echocardiographic plus two minor electrocardiographic criteria according to McKenna et al. [23].

Individuals with prior cardiac surgery (including septal myectomy), alcohol septal ablation, coronary artery disease, neoplasms, liver disease, chronic renal failure or any other severe systemic disease were not included in the present study. Significant atherosclerotic coronary artery disease (>50% stenosis in a major coronary artery) was defined on the basis of clinical (acute coronary event associated with cardiac enzymes increase or Q waves on ECG) or cardiac magnetic resonance (CMR) LGE pattern.

Written informed consent was obtained from each patient included in the study. The study was approved by the Ethical Committee of the Fondazione Policlinico Tor Vergata (Rome, Italy), in accordance to the principles of the Declaration of Helsinki.

### Ecocardiography

Two-dimensional echocardiography (Vivid E9 GE-Healtcare, Horten, Norway) was performed to assess left ventricular hypertrophy and the site and extent of maximal wall thickness were identified. As the enrolled HCM subjects shared the preferential IVS localization of hypertrophy, maximum end-diastolic IVS thickness was taken as the greatest LV wall dimension. Left ventricular outflow obstruction, due to mitral valve systolic anterior motion and mitral-septal contact, was identified by a peak instantaneous outflow gradient ≥30 mmHg under basal conditions (resting obstruction) or by a dynamic gradient ≥30 mmHg during expiratory effort (provokable outflow obstruction) [20] with continuous-wave Doppler.

### Cardiac Magnetic Resonance (CMR)

**Fig 1:**
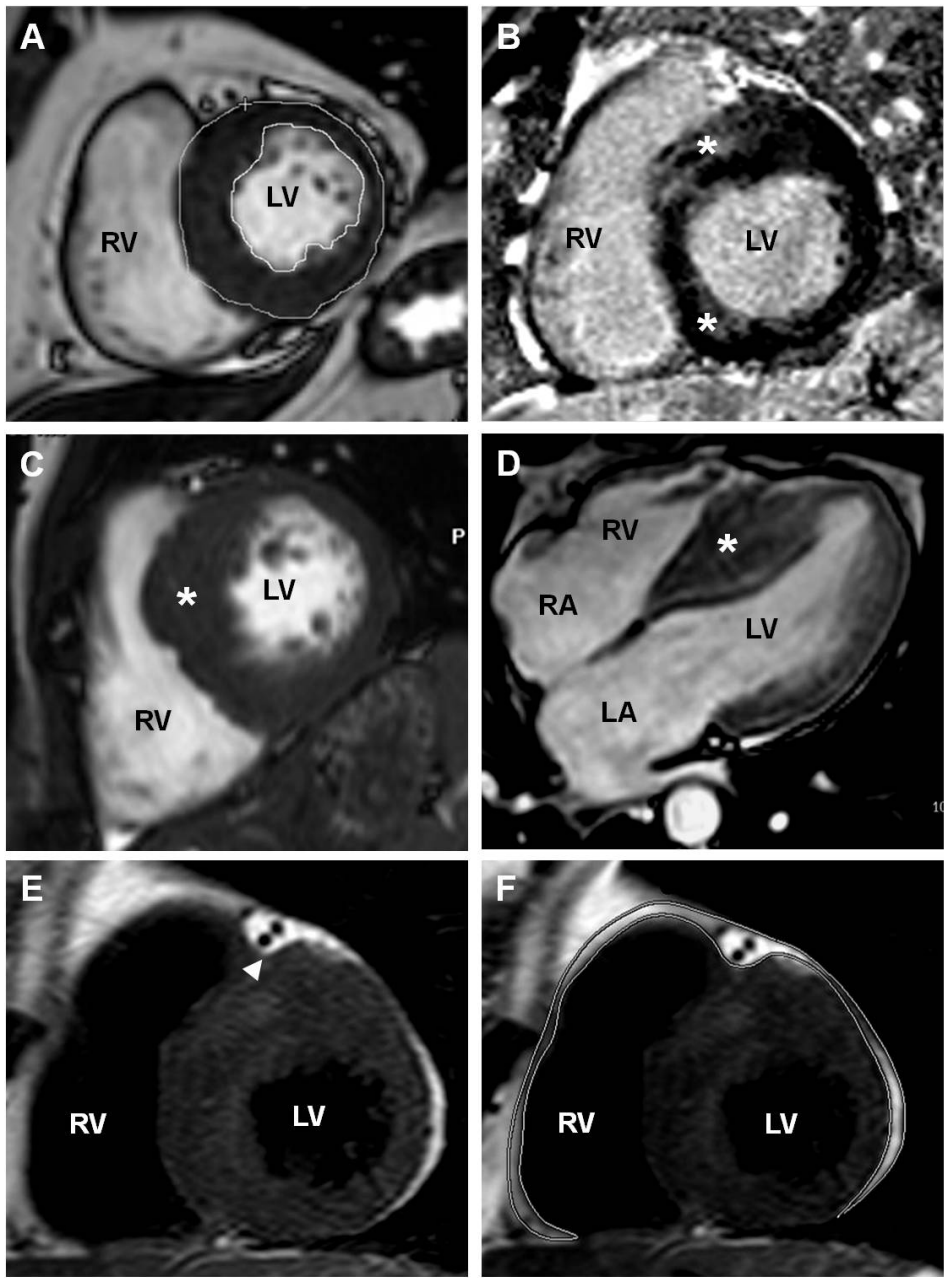
CMR measurements in patients with HCM. (A) LV mass was obtained tracing manually the endocardial and epicardial borders of the LV on successive short-axis cine images at end-diastole. LV mass was then derived by the summation of discs method and multiplying myocardial muscle volume by 1.05 g/cm^3^. (B) LGE assessment. LGE (asterisk) appears prevalent in regions of hypertrophy, mainly in a patchy, multifocal mid-wall distribution. (C-D) Asymmetric LV hypertrophy. CMR short axis (2-chamber) (C) and horizontal long axis (4-chamber) (D) view showing marked IVS thickening (asterisk); (E) EAT thickness measured at the anterior interventricular groove site (arrowhead). (F) Volumetric assessment of EAT. The contours of EAT were outlined in end-diastolic images of short-axis views covering the whole left and right ventricle. The areas obtained for each slice were added together and multiplied by slice thickness to yield EAT volume. Abbreviations: CMR, cardiac magnetic resonance; EAT, epicardial adipose tissue; HCM, Hypertrophic Cardiomyopathy; IVS, interventricular septum; LGE, late gadolinium enhancement; LV, left ventricle; RV, right ventricle.

CMR examinations were performed using a commercially available scanner (Philips Intera 1.5 Tesla Achieva, Eindhoven, The Netherlands) within 1 month from echocardiographic evaluation. ECG-gated, steady-state, free precession breath-hold cines in sequential 10-mm short-axis slices (no gap) were acquired starting parallel to the atrioventricular ring and covering the entire ventricle. LV and RV end-diastolic and end-systolic volumes, stroke volumes and ejection fraction (EF), LV mass and wall thickness were measured off-line using a stand-alone work station (Extended MR WorkSpace 2.6.3.4, 2012 Philips Medical System) (Fig 1). For calculation of LV mass, the endocardial and epicardial borders of the LV were manually planimetered on successive short-axis cine images at end-diastole. Particular care was taken to avoid including papillary muscles in the LV mass calculation. LV mass was derived by the summation of discs method and multiplying myocardial muscle volume by 1.05 g/cm^3^ [21]. LV mass and ventricular end-diastolic and end-systolic volumes were indexed to body surface area. The presence of LGE, the imaging biomarker of cardiac fibrosis, was assessed by visual inspection 15 min after intravenous administration of 0.1 mmol/kg gadobutrol (Gadovist, Bayer, Berlin, Germany) with breath-hold segmented inversion recovery sequence, which was acquired in the same views as the cine images (Fig 1). Moreover, all patients underwent a standardized protocol including quantification of EAT volume during CMR (Fig 1). For the assessment of EAT, we used a black blood prepared T2-weighted multislice to obtain a transversal 4-chamber view and short-axis images. Images parameters were as follows: time of repetition (TR) = 1600 ms, time to echo (TE) = 70 ms, slice thickness = 4 mm, interslice gap (GAP) = 2 mm and field of view (FOV) = 450 mm. EAT only included fat between the myocardial border and the internal visceral layer of the pericardium. Areas of EAT were traced manually on consecutive end-diastolic short-axis images beginning at the mitral valve and ending at the last slice containing cardiac tissue. The areas obtained for each slice were added together and multiplied by slice thickness to yield EAT volume [24, 25].

CMR measurements were performed by an experienced investigator, blinded to the results of echocardiography.

### Clinical and anthropometric assessment

Blood pressure (BP) was measured in the sitting position, with a standard, appropriately sized sphygmomanometer cuff. Systemic hypertension was diagnosed based on resting blood pressure values >140/90 mmHg on three different examinations or anti-hypertensive treatments [26].

Cardiac functional status was assessed and classified according to the New York Heart Association (NYHA) functional classes (I, II, III, or IV), on the basis of patient's limitations in physical activities caused by cardiac symptoms [27]. Waist circumference was recorded as the average of two measurements while the patients were standing, at midpoint between the lowest rib and the iliac crest. Body mass index (BMI) was calculated by dividing the weight (in Kg) by the square of height (in meter).

Biochemical tests (blood count, urea, creatinine, electrolytes, total protein, glucose, insulin, total-, High Density Lipoprotein (HDL)- and Low Density Lipoprotein (LDL)-cholesterol, triglycerides, alanine and aspartate aminotransferase, gamma-glutamyl transferase, uric acid, N-terminal prohormone of brain natriuretic peptide (NT-proBNP), high sensitivity C-Reactive Protein (hsCRP) and erythrocyte sedimentation rate (ESR) of blood samples obtained after overnight fasting were assessed by routine laboratory techniques. The homeostasis model assessment (HOMA) index was calculated as fasting insulin (mU/mL) × fasting glucose (mg/dl)/405 [28]. Metabolic syndrome (MS) diagnosis was made using National Cholesterol Education Program (NCEP) Adult Treatment Panel III (ATP III) criteria [29].

### Dual energy X-ray absorptiometry (DXA)

Body composition and regional fat distribution were assessed by Dual-energy X-ray absorptiometry (DXA) [30] (DelphiWscanner, Hologic Co., Bedford, MA). The scans were analyzed using Hologic Discovery software (version 12.2) with manual inspection of regions of interest.

Body composition estimates including total body fat mass (g) (TB-FAT) and lean mass (bone free) (g) were obtained from the total body DXA scan. Percentage TB-FAT mass was calculated as (fat mass/total mass) × 100 and fat and lean mass indexes were calculated as fat mass (kg)/height (m^2^) and lean mass (kg)/height (m^2^) respectively. Trunk fat mass (Tr-FAT) was also distinguished from peripheral or appendicular (upper and lower limbs) fat mass as a measure of abdominal adiposity. Tr-FAT was defined by the region below the chin, delineated by vertical lines within the left and right glenoid fossae bordering laterally to the ribs and by the oblique lines that cross the femoral necks and converge below the pubic symphysis. Percentage Tr-FAT was calculated as (trunk fat mass/total mass) ×100 and Tr-FAT index was calculated as trunk fat mass (Kg)/height (m^2^). Annual servicing and calibration according to manufacturer's specifications were carried out and calibration of the DXA machine using a phantom was performed prior to each scanning session.

### Statistical analysis

Descriptive statistics were given by means±SD or median (interquartile range), as appropriate. The Kolmogorov-Smirnov test was used to verify quantitative variables for normality distribution, and skewed variables (diastolic BP, glucose, aminotransferases, hsCRP, NT-proBNP, indexed upper limb FAT, left atrium (LA) area and LA diameter) were logarithmically transformed before being used in the subsequent parametric procedures. The appropriateness of these log-transformation was confirmed by subsequent Kolmogorov-Smirnov test for all the transformed variables (p>0.05 for all). Comparisons between groups (males vs. females; MS vs. noMS; hypertensive subjects vs. non-hypertensive subjects; LGE positive vs. LGE negative subjects) were made using Student’s unpaired t-test. Clinical features independently associated with maximum IVS thickness, LV mass index, LV ejection fraction (LVEF), RV ejection fraction (RVEF) and NIHA functional class were assessed by multivariate regression analysis, including gender and age as potential confounders. Resulting β coefficients were provided to evaluate the strengths of the associations. For all these analysis a p-value <0.05 based on two-sided test was considered statistically significant. Statistical analysis was performed with the SPSS 19.0 software (SPSS, Chicago).

## Results

### HCM phenotype and gender

As displayed in Table 1, women presented significantly higher percentages of total fat mass compared with men (M: 30.4±5.8%; F: 38.3±6.3%, p = 0.007). Similar differences were seen for both lower limbs fat (M: 26.6±6.6%; F: 40.4±5.5%, p = 0.0001) and upper limbs fat (M: 30.6±8.6%; F: 44.9±11.9%, p = 0.007).

**Table 1:** Gender-stratified characteristics of the study population.

	Overall	F	M	*p*
**CLINICAL FEATURES**				
**N**	32	10	22	
**Age (y)**	57.2±12.6	62.9±10.3	54.7±12.9	*ns*
**BMI (Kg/m**^**2**^**)**	29.9±3.9	29.4±4.2	30.1±3.9	*ns*
**SBP (mmHg)**	134.3±15	135±14.7	134±15.5	*ns*
**DBP (mmHg)**	80 (70–85)	80 (67.5–82.5)	80 (77.5–86.2)	*ns*
**Hypertension**	16	6	10	
**Atrial fibrillation**	3	2	1	
**Smokers**	9	1	8	
**T2D**	4	3	1	
**Fasting glucose (mg/dl)**	105.2±22.6	107±19.1	104.4±24.3	*ns*
**Insulin (mU/mL)**	11.3±4	11.8±4.8	11.2±3.8	*ns*
**HOMA-IR**	2.8±1	3±1.2	2.8±1	*ns*
NYHA class (I/2/3/4)*	13/13/6/0	3/5/2/0	10/8/4/0	
**NT-proBNP (pg/ml)**	228.5 (120–604.7)	294.3 (169.5–1312)	171 (117.9–487.5)	*ns*
**On medical treatment**:				
**Beta-blockers**	28	9	19	
**Verapamil**	2	1	1	
**Disopyramide**	2	1	1	
**ACE-inhibitors/ARBs**	16	6	10	
**DXA FINDINGS**				
**TB-FAT (%)**	32.9±6.9	38.3±6.3	30.4±5.8	*0*.*007*
**Tr-FAT (%)**	34.7±6.6	37.3±7.6	33.6±6	*ns*
**Lower limb FAT (%)**	30.9±8.9	40.4±5.5	26.6±6.6	*0*.*0001*
**Upper limb FAT (%)**	35±11.6	44.9±11.9	30.6±8.6	*0*.*007*
**ECHOCARDIOGRAPHIC FINDINGS**				
**LA area (cm**^**2**^**)**	25 (23–29)	25 (23–27)	25 (23–29)	*ns*
**LA diameter**	44 (40.5–48)	43 (33–47)	44.5 (40.5–49.7)	*ns*
**LV end-diastolic diameter**	45.4±4.9	45.2±6.1	45.5±4.4	*ns*
**LV outflow obstruction (n)**	9	2	7	
**CMR FINDINGS**				
**EAT total V (ml)**	71.2±20.3	77.1±31	68.5±14.4	*ns*
**EAT thickness**	16.3±3.2	14.8±3.4	17±2.9	*ns*
**Max IVS thickness**	17±4.3	14.3±4	18.3±3.8	*0*.*01*
**LV mass (g/m**^**2**^**)**	82.9±26.1	72.8±30.8	87.3±23.5	*ns*
**LVEF (%)**	71.2±7.9	70.6±10.2	71.5±6.8	*ns*
**LV Stroke V (ml)**	79.7±16.3	74±11.9	81.1±17.6	*ns*
**LV end-diastolic V (ml/m**^**2**^**)**	56.7±29.9	57.2±9.9	56.5±10.3	*ns*
**LV end-systolic V (ml/m**^**2**^**)**	16.1±6	16.5±8.3	16±5	*ns*
**RVEF (%)**	63.2±7.1	67.5±6.3	61.3±6.7	*0*.*048*
**RV Stroke V (ml)**	77±18.3	67±10.1	81.3±19.6	*0*.*03*
**RV end-diastolic V (ml/m**^**2**^**)**	60.5±17	90.1±27.8	132.5±32	*0*.*04*
**RV end-systolic V (ml/m**^**2**^**)**	22±8.6	31.8±14.4	51±17	*0*.*04*
**LGE (n)**	17	3	14	

ARBs, Angiotensin receptor blockers; BMI, body mass index; CMR, cardiac magnetic resonance; DBP, diastolic blood pressure; DXA, dual energy X-ray absorptiometry; EAT, epicardial adipose tissue; ESR, erythrocyte sedimentation rate; F, females; HOMA, homeostatic model assessment; IVS, interventricular septum; LA, left atrium; LGE, late gadolinium enhancement; LV, left ventricle; LVEF, left ventricle ejection fraction; M, males; NYHA, New York Heart Association; RV, right ventricle; SBP, systolic blood pressure; T2D, type 2 diabetes; TB, total body; Tr, trunk; V, volume.

* at most recent evaluation. Data are expressed as mean ± SD; log-transformed variables (DBP, NT-proBNP, LA area, LA diameter) are expressed as median (interquartile range). P value refers to F vs. M comparisons (unpaired t-test); ns, not significant.

Average maximum IVS thickness in our group was 17±4.3 mm (ranging from 8 to 27 g/m^2^), with greater values observed in males (M: 18.3±3.8 mm vs. F: 14.3±4 mm, p = 0.012) (Table 1). Average LV mass index turned out to be greater in male gender as well (M: 87.3±23.5 g/m^2^; F: 71.8±30.8 g/m^2^), although not statistically significant (Table 1). With respect to male gender, we also observed lower RV systolic function, as expressed by RVEF (M: 61.3±6.7%; F: 67.5±6.3%, p = 0.048), and higher indexed RV end-diastolic (M: 64.8±16.3 ml/m^2^; F: 50.7±15.5 ml/m^2^, p = 0.04) and end-systolic volumes (M: 24.3±8.3 ml/m^2^; F: 16.7±7.4 ml/m^2^, p = 0.04). Therefore, male patients showed greater indexed RV cavity size compared to women, even if, average RV end-diastolic dimension remained within the normal range for both groups, entailing a nondilated RV cavity. By virtue of greater end-diastolic volumes, stroke volume of the RV was also increased in males (M: 81.3±19.6 ml; F: 67±10.1 ml, p = 0.03).

Differently, LV volumes and function (as expressed by LV end-diastolic volume index, LVEF and LV stroke volume) were virtually identical in men and women.

### HCM phenotype and metabolic syndrome

We compared regional adiposity and cardiac parameters of subjects affected by metabolic syndrome with those of subjects who were not (MS: n = 15; noMS: n = 17). The same analysis was performed to compare hypertensive patients (n = 16) with those non-hypertensive (n = 16) at the moment of HMC diagnosis. In both cases, we have not found any difference in adiposity distribution and cardiac morphology and function. Only, patients with MS showed significantly greater percentage TB-FAT (MS: 35.5±4.8%; noMS: 30.4±7.8%, p = 0.04) and epicardial fat total volume (MS: 83.6±20.9 ml; noMS: 58.8±9.7 ml, p = 0.01).

### HCM phenotype and regional adiposity

Multivariate regression analysis was performed to identify regional fat depots independently associated with hypertrophy extent (defined by maximum IVS thickness, LV mass index) and clinical status (LVEF and NYHA class at the most recent evaluation). Variables assessed included total adiposity (BMI or percentage TB-FAT) regional fat distribution measures, age and sex (Table 2). Among them, only maximum IVS thickness (p = 0.02), but not LV mass index, resulted positively associated to Tr-FAT independent of age and gender (Table 2), with TB-FAT showing a similar, but not significant, trend (p = 0.07). By contrast, no relationships of cardiac parameters with appendicular fat and EAT were observed. Of note, EAT total volume was related with NT-proBNP levels (β = 0.63, p = 0.04) (Table 2). As expected, higher BMI was associated with increasing NYHA class (p = 0.01).

**Table 2:** Age- and gender-adjusted associations between hypertrophy extent, NYHA class and adiposity distribution measures.

	Maximum IVS thickness		LV mass index		LVEF		NYHA class*	
	*Age- and gender-adjusted*							
	*β*	*p*	*β*	*P*	*β*	*p*	*β*	*p*
**BMI (Kg/m**^**2**^**)**	0.05	*0*.*97*	0.24	*0*.*28*	0.03	*0*.*98*	**0.49**	***0*.*01***
**TB-FAT (%)**	0.37	*0*.*07*	0.38	*0*.*14*	-0.01	*0*.*95*	0.44	*0*.*08*
**Tr-FAT (%)**	**0.43**	***0*.*02***	0.26	*0*.*3*	0.23	*0*.*33*	0.36	*0*.*11*
**Lower limb FAT (%)**	0.26	*0*.*30*	0.38	*0*.*17*	-0.17	*0*.*56*	0.44	*0*.*12*
**Upper limb FAT(%)**	0.17	*0*.*42*	0.38	*0*.*13*	-0.25	*0*.*32*	0.35	*0*.*17*
**EAT total V (ml)**	0.20	*0*.*56*	-0.15	*0*.*64*	-0.15	*0*.*65*	0.04	*0*.*90*

BMI, body mass index; EAT, epicardial adipose tissue; IVS, interventricular septum; LV, left ventricle; LVEF, left ventricle ejection fraction; NYHA, New York Heart Association; TB, total body; Tr, trunk; V, volume.

* at most recent evaluation. Statistically significant associations (p<0.05) are highlighted in bold.

**Fig 2:**
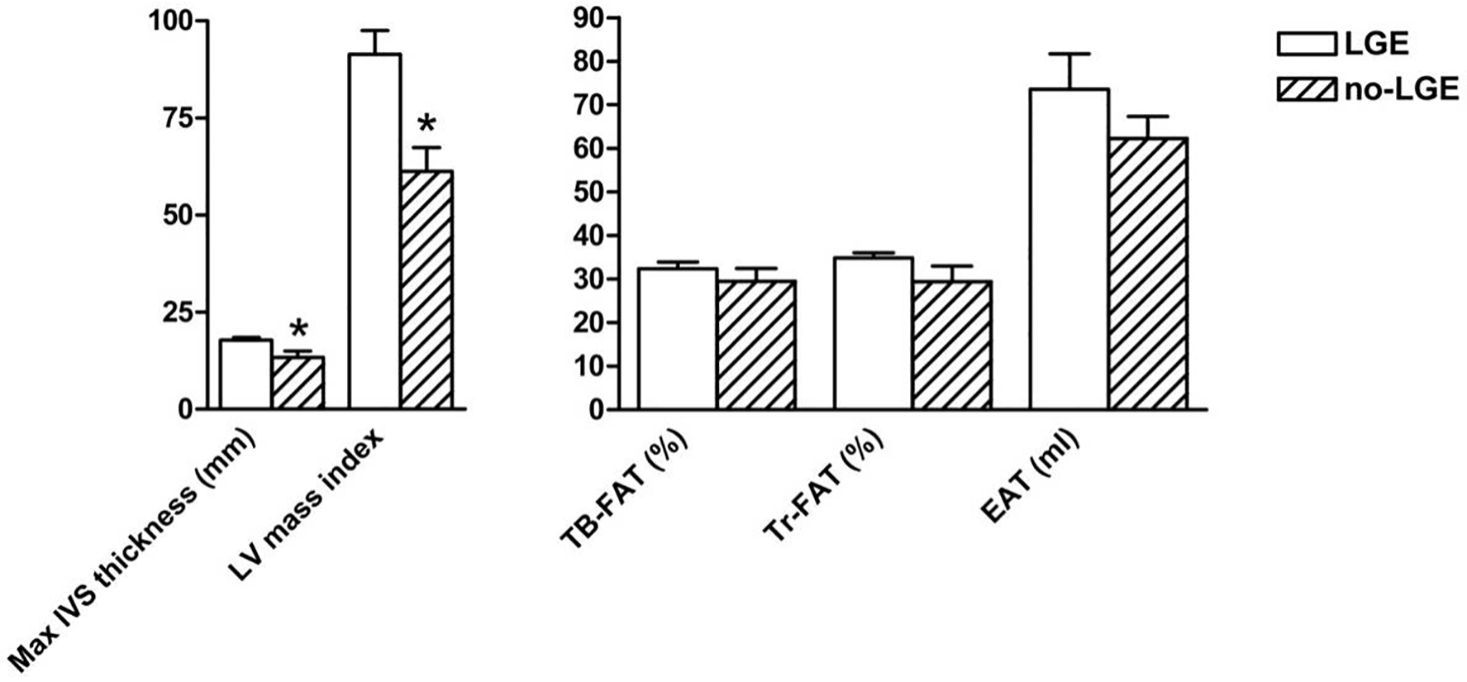
Cardiac parameters on CMR and regional fat distribution in subjects with and without LGE. Abbreviations: CMR, cardiac magnetic resonance; EAT, epicardial adipose tissue; IVS, interventricular septum; LGE, late gadolinium enhancement; LV, left ventricle; TB, total body; Tr, truncal. Data are presented as mean±SD. * p<0.05.

Seventeen patients (3F/14M) of our cohort were positive to LGE assessment on CMR as shown in Table 1. Compared to LGE negative subjects, they displayed greater maximum IVS thickness (17.8±3 vs. 13.5±3.8 mm, p = 0.02), LV mass index (91.3±24.4 vs. 63.5±19.6 g/m^2^, p = 0.015) and NT-proBNP serum levels (335 [131–1100] vs. 221 [58–439] pg/ml, p = 0.04), but no relationships either with fat amount or distribution were observed (Fig 2).

## Discussion

The present study showed that in our HCM cohort maximum IVS thickness, but not LV mass index, was independently associated to abdominal adiposity, not showing any relationship with appendicular fat, a surrogate estimate of subcutaneous fat. NT-proBNP levels resulted independently associated with EAT amount. Subjects displaying regions of myocardial LGE, which represents regions of increased myocardial collagen and adverse ventricle remodeling, showed greater maximum IVS thickness, LV mass index and NT-proBNP levels, but no relationship with fat amount or distribution. Finally, our data confirmed that male gender represents an important factor strongly associated to the magnitude of hypertrophy.

The major strengths of the present study were the phenotypically homogeneous cohort of HMC patients with preferential localization of hypertrophy at the IVS, and the use of a highly reliable CMR-based assessment of cardiac morphology and function. CMR also provides the most accurate quantification of EAT deposit. Indeed, a volumetric measurement of EAT using CMR is known to be less influenced by individual cardiac anatomy and fat distribution compared with echocardiographic measurements at single points [31]. In addition to EAT total volume, EAT thickness was measured at the anterior interventricular groove as the nearest site to the hyperthophic IVS, based on the notion that EAT could exert paracrine effects on the contiguous myocardium [14]. At the same time, we evaluated body regional (trunk and appendicular) fat distribution by means of DXA.

To our knowledge, the present study represents the first report of an association between abdominal adiposity (as measured by Tr-FAT) and the phenotypic expression of LV hypertrophy in HMC.

In the general population, LV remodeling associated with chamber enlargement is an established consequence of obesity, reflecting a physiologic adaptation to body weight gain [32], so that obesity is considered an important predictor of heart failure [33].

HMC, which has historically been considered a mere consequence of gene mutation despite the morphologic heterogeneity even within the same family [3, 34, 35], also implies an environmental modulation. Indeed, greater LV mass has been observed in male patients and in those with dynamic outflow obstruction [21] or systemic hypertension [26, 36]. In addition, in two recent reports on HMC, BMI was associated with heart failure symptoms progression as well as increased LV mass index but not septal wall thickness [10, 37].

Even if we confirmed that increased BMI is related to heart failure symptoms progression (as expressed by NYHA class), by negatively impacting the cardiovascular system and viciously obliging to a sedentary lifestyle, it is neither a reliable marker of fat accumulation (38) nor an indicator of fat distribution. Therefore, we extended these previous observations by providing an estimate of fat distribution and extent and found that central adiposity (Tr-FAT) is associated to maximum IVS thickness, the hallmark feature most differentiating HCM from secondary forms of LV hypertrophy (34). Although our findings are necessarily restricted to the population included in our study with its intrinsic clinical features and need to be confirmed on a larger population, they support the hypothesis that abdominal fat distribution, in spite of BMI and subcutaneous fat, may be a factor impacting on HCM cardiac phenotype. Abdominal adiposity seems more strongly related to LV remodelling and adverse hemodynamics than subcutaneous fat [16], putatively acting on HCM-distinctive LV regional thickening by modulating the genetic etiology (e.g. by inducing further stress, abnormal protein production and accumulation) [38]. Alternatively, it might act through the same hemodynamic, neuro-hormonal and oxidative pathways of secondary hypertrophy [33], especially impacting the ventricular wall where the thickening is primitively more pronounced.

In contrast, we did not find associations between fat distribution and LV mass index. After all, increased LV mass is not invariably present in patients with HCM [39], whereas increased regional ventricular wall thickness is the real hallmark of the LV hypertrophy in HCM, which is indeed typically asymmetric [7].

Although EAT accumulation has been related to abdominal fat [25], myocardial mass [13] and, in our cohort, to serum NT-proBNP, a generally accepted marker of heart failure, it did not correlate with the hypertrophy pattern. This finding supports the hypothesis that the association between Tr-FAT and IVS thickness is mainly systemically mediated, rather than locally by EAT. Nevertheless, the dynamic modification in LV geometry coming along with LV remodelling due to progressive heart failure may, at least in part, account for our finding as much as for the reversal of the expected correlation between LV hypertrophy and EAT measures [40].

As myocardial fibrosis may provide the underlying arrhythmogenic substrate in HMC, there has been growing interest in evaluating LGE on CMR as an independent predictor of adverse cardiac outcomes in HMC [41]. Indeed, LGE, which is highly prevalent in regions of hypertrophy, mainly in a patchy, multifocal mid-wall distribution [42], has been shown to be associated with non-sustained ventricular tachycardia as well as with other risk factors for sudden cardiac death [43, 44]. In keeping with this, in our cohort, patients who were LGE positive displayed significantly greater maximum IVS thickness, LV mass and NT-proBNP concentrations.

EAT may play a pathogenetic role in heart dysfunction by secreting pro-fibrotic factors such as Activin A (known to be involved in heart failure pathogenesis) [14, 45] and cardio-depressant mediators such as FABP4 [46], by promoting cardiomyocyte apoptosis through local increase of intra-myocardial fatty acids, oxidative stress and inflammation [47].

Even though EAT amount was not significantly greater in LGE positive patients, it is tempting to speculate that LGE may be associated to a, if not increased, more metabolically active EAT. Indeed, the paracrine pro-fibrotic properties of EAT on neighbouring myocardium [14] and its association with AF and remodelling in HMC are highly suggestive [31].

Maximum IVS thickness was substantially greater in male than female patients, in accordance with CMR-calculated LV mass index [21]. Nevertheless, this finding contrasts with prior echocardiographic studies which reported little difference between the genders in maximal wall thickness [39, 48].

Finally, we acknowledge some study limitations, mainly related to the small sample size that did not allow either to perform further subgroup analysis or adjustments beyond gender (e.g. for hypertension and diabetic status). Indeed, hemodynamic and neuro-hormonal abnormalities associated to hypertension [49] [50] and type 2 diabetes-related metabolic derangements such as hyperglycaemia, lipotoxicity, and hyperinsulinaemia [51] may influence LV mass in HMC patients. Nevertheless, the hypertensive patients of our cohort were all pharmacologically treated and the number of diabetics extremely exiguous (n = 4) (even though the actual prevalence of diabetes may have been underestimated by the diagnosis exclusively based on clinical history and serum fasting glucose). However, subjects diagnosed with MS or hypertension (the latter at the moment of HMC diagnosis) did not show significant differences in cardiac parameters, not even differing by regional adiposity. We also recognize the lack of LGE quantification which could have been useful to evaluate the relation between myocardial fibrosis and EAT amount. Finally, the morphologically homogeneous sample of HMC patients, who indeed were selected on the basis of the preferential localization of hypertrophy within the IVS, did not allow to explore the association between fat distribution and the phenotypic variability of HMC.

In conclusion, the present study provides evidence that only central adiposity, but not appendicular or epicardial fat, affects the magnitude of cardiac regional hypertrophy in patients with HCM. We also confirmed that male gender represents another important extrinsic factor affecting the severity of HCM phenotype.
